# Cancer

**Published:** 1883-10

**Authors:** 


### CANCER.

CHIEN TURPENTINE has been recommerded as a cure for cancer; but it seems that its usefulness is limited to allaying the pain incident to this unfortunate disease. An old and esteemed friend of ours manifests a kindness worthy of all commendntion in his desire to make as widely known as possible the result of his observation in the use of this remedy• His mother has cancer, and the son, during the two or three years she has been afflicted, has left no remedy untried that offered the slightest promise of benefit. Among the remedies tried was CHIEN TURPENTINE. It was given in pills 2 or 3 times a day, so that the amount taken each day should amount to 18 or 20 grains. It acted like magic. The pain diminished or disappeared, and the march of the disease seemed to be checked, so that hopes of cure were indulged in. These hopes, however, proved vain, for the progress of the disease, though slow, was not arrested; and the remedy was discontinued. The pain soon returned after the medicine was stopped; but it again ceased when the medicine was resumed. Several trials of this sort were made, and our friend feels confident that if the remedy has not the power to cure the disease, it has the power to impede its progress and to give the patient almost entire immunity from suffering.

A medicine that can accomplish so desirable an end should be widely known, and it is to be hoped that "the press" will aid us in bringing it into general notice. Chien Turpentine is an article of commerce, and is to be found at almost any drug store.

